# Ethnic Differences in the Frequency of *CFTR* Gene Mutations in Populations of the European and North Caucasian Part of the Russian Federation

**DOI:** 10.3389/fgene.2021.678374

**Published:** 2021-06-16

**Authors:** Nika Petrova, Natalia Balinova, Andrey Marakhonov, Tatyana Vasilyeva, Nataliya Kashirskaya, Varvara Galkina, Evgeniy Ginter, Sergey Kutsev, Rena Zinchenko

**Affiliations:** ^1^Research Centre for Medical Genetics, Moscow, Russia; ^2^N.A. Semashko National Research Institute of Public Health, Moscow, Russia

**Keywords:** allelic polymorphism, *CFTR* gene variants, population differences, ethnic diversity, European and North Caucasian part of Russia

## Abstract

Cystic fibrosis (CF) is a common monogenic disease caused by pathogenic variants in the *CFTR* gene. The distribution and frequency of *CFTR* variants vary in different countries and ethnic groups. The spectrum of pathogenic variants of the *CFTR* gene was previously studied in more than 1,500 CF patients from different regions of the European and North Caucasian region of Russia and the spectrum of the most frequent pathogenic variants of the *CFTR* gene and ethnic features of their distribution were determined. To assess the population frequency of *CFTR* gene mutations some of the common variants were analyzed in the samples of healthy unrelated individuals from the populations of the European part of the Russian Federation: 1,324 Russians from four European regions (Pskov, Tver, Rostov, and Kirov regions), representatives of five indigenous ethnic groups of the Volga-Ural region [Mari (*n* = 505), Udmurts (*n* = 613), Chuvash (*n* = 780), Tatars (*n* = 704), Bashkirs (*n* = 517)], and six ethnic groups of the North Caucasus [Karachay (*n* = 324), Nogais (*n* = 118), Circassians (*n* = 102), Abazins (*n* = 128), Ossetians (*n* = 310), and Chechens (*n* = 100)]. The frequency of common *CFTR* mutations was established in studied ethnic groups. The frequency of F508del mutation in Russians was found to be 0.0056 on average, varying between four regions, from 0.0027 in the Pskov region to 0.0069 in the Rostov region. Three variants W1282X, 1677delTA, and F508del were identified in the samples from the North Caucasian populations: in Karachay, the frequency of W1282X mutation was 0.0092, 1677delTA mutation – 0.0032; W1282X mutation in the Nogais sample – 0.0127, the frequency of F508del mutations was 0.0098 and 1677delTA – 0.0098 in Circassians; in Abazins F508del (0.0039), W1282X (0.0039) and 1677delTA (0.0117) mutations were found. In the indigenous peoples of the Volga-Ural region, the maximum frequency of the F508del mutation was detected in the Tatar population (0.099), while this mutation was never detected in the Mari and Bashkir populations. The E92K variant was found in Chuvash and Tatar populations. Thus, interethnic differences in the spectra of *CFTR* gene variants were shown both in CF patients and in healthy population of the European and North Caucasian part of Russia.

## Introduction

Cystic fibrosis (CF; OMIM 219700) is a common monogenic disease caused by a mutation of the *CFTR* gene (CFTR, OMIM 602421; reference sequence accession number NM_000492.3). To date, more than 2,100 variants of the *CFTR* gene have been identified (Cystic Fibrosis Mutation Database, 2011), the distribution and frequency of which vary in different regions and ethnic groups (Bobadilla et al., 2002; Lao et al., 2003; Schrijver, 2011; World Health Organization [WHO], 2021]. In CF patients the most common mutations are F508del (66.8%), G542X (2.6%), N1303K (1.6%), G551D (1.5%), W1282X (1.0%), 1717-1G → A (0.83%), R553X (0.75%), 621 + 1G → T (0.54%), and R1162X (0.51%) (Estivill et al., 1997; World Health Organization [WHO], 2021). There is a decreasing proportion of CF patients with F508del from northwestern to southeastern Europe (Lucotte and Hazout, 1995; Bobadilla et al., 2002; Atag et al., 2019; Farrell et al., 2018), the highest frequency in Denmark (87.2%) and the lowest in Algeria (26.3%). Mutation G542X is common in the Mediterranean countries (6.1%). N1303K is found in most of the western and Mediterranean countries with the highest frequency in Tunisia (17.2%). G551D is common in north-west and central Europe. W1282X has the highest frequency in Israel (36.2%), being also common in most Mediterranean countries and North Africa (Estivill et al., 1997; Bobadilla et al., 2002; Lao et al., 2003; World Health Organization [WHO], 2021).

**FIGURE 1:**
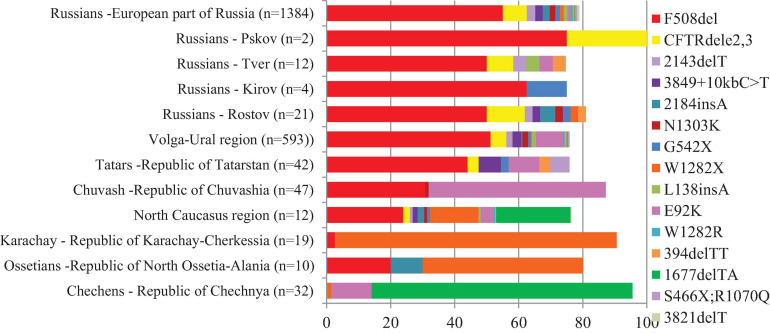
Relative frequencies of common *CFTR* variants in studied regions and ethnic groups of European part and North Caucasus of Russia.

The population of the European part of Russia, represented by more than 70 ethnic groups, is about 109 million people. Previously, we studied the spectrum of pathogenic variants of the *CFTR* gene in more than 1,500 CF patients living in different regions of the European part of Russia and determined the spectrum of the most common pathogenic variants of the *CFTR* gene and the ethnic features of their distribution (Stepanova et al., 2012; Petrova et al., 2016, 2020a; Petrova N. V. et al., 2019). The difference in the spectra of the *CFTR* gene pathogenic variants in CF patients in different populations of Russia was shown. For ethnic Russian CF patients, a significant diversity of the spectrum of *CFTR* variants was shown: up to 98% of mutant alleles were caused by 110 variants, the most common were F508del (55%), CFTRdele2,3 (7.5%), 2143delA (2.7%), 3849 + 10kbC-T (2.3%), 2184insA (2.2%), N1303K (1.7%), G542X (1.5%), W1282X (1.2%), L138ins (1.1%), E92K (1.0%), and W1282R (0.7%) (Petrova et al., 2020a). The low diversity of the *CFTR* gene variant spectra was revealed in the ethnic groups of the North Caucasus region. The W1282X variant accounted for 88% of Karachays (Petrova et al., 2016), the high proportion of 1677delTA (81.5%) and E92K (12.5%) variants in Chechens (Petrova N. V. et al., 2019), and W1282X (50%) and F508del (20%) variants in Ossetians [Petrova et al., 2020b (in Russ.)] were found. The prevalence of E92K (55%) and F508del (30%) variants was noted among Chuvash, one of the ethnic groups of the Volga-Ural region (Stepanova et al., 2012). The distribution of relative frequencies of common *CFTR* variants in studied populations were shown in Figure 1 (Supplementary Table 1).

Data on the mutation spectrum and prevalence of major *CFTR* gene mutations in various ethnic groups are important for the development of molecular diagnostics tools for identifying genetic causes of CF; however, data on the prevalence of common *CFTR* mutations in some populations are still not available.

Here, we present data on frequencies of major *CFTR* mutations in Russian Federation, in 15 populations of European Russia and North Caucasus.

## Materials and Methods

**FIGURE 2:**
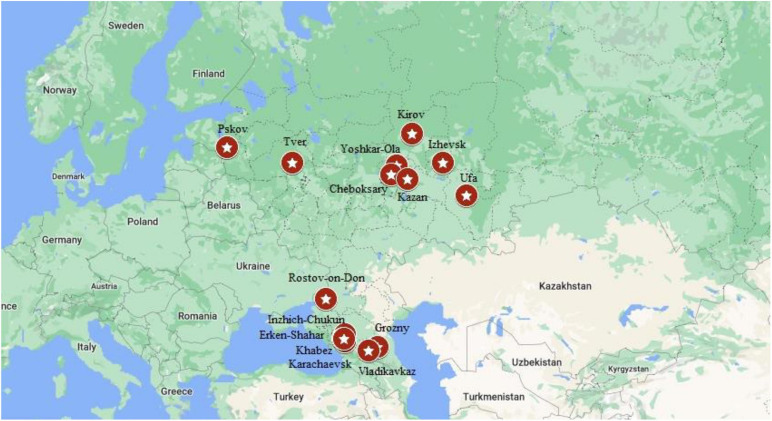
Location of studied populations. Russians – Pskov, Tver, Kirov, Rostov-on-Don. Volga-Ural region: Yoshkar-Ola – Mari; Cheboksary – Chuvashes; Kazan – Bashkirs; Izhevsk – Udmurts; Ufa – Bashkirs. North Caucasus region: Inzhich-Chukum – Abaza; Erken-Shakar – Nogais; Khabez – Circassians; Karachaevsk – Karachay; Vladikavkaz – Ossetians; Grozny – Chechens.

The total number of 5505 DNA samples of healthy unrelated individuals – representatives from 15 various populations of 12 ethnic groups living in the territory of European Russia have been studied. These are Russians (648 from Rostov, 354 – from Kirov, 182 – from Tver and 140 – from Kirov regions), Udmurts (*N* = 613), Maris (*N* = 505), Bashkirs (*N* = 517), Tatars (*N* = 704), Chuvashes (*N* = 780), Karachays (*N* = = 324), Nogais (*N* = 118), Cherkessians (*N* = 102), Abaza (*N* = 128), Chechens (*N* = 100), and Ossetians (*N* = 310). The mean age was 31 years (range 18–65), gender ratio – 0.40 males: 0.60 females. None of them had discernable symptoms suggestive of CF. The places of location of the populations under study are shown in Table 1 and Figure 2.

**TABLE 1:** Allele frequencies of identified mutations in studied populations of European part of Russia (Eastern European, Volga-Ural and North Caucasus regions).

**Ethnic group (region)**	**Linguistic family/group**	**Sample size**	**Mutant/tested chromosomes frequencies (95% CI)**					
			**F508del**	**CFTRdele2,3**	**1677delTA**	**E92K**	**L138ins**	**W1282X**
Russians (European part of Russia – 4 regions)	Indo-European/Slavic	1,324	15/2648 0.0057 (0.0032–0.0093)	1/2648 0.0004 (0.0000–0.0021)	1/2648 0.0004 (0.0000–0.0021)	0/810 0.0000 (0.0000–0.0037)	0/810 0.0000 (0.0000–0.0037)	1/890 0.0011 (0.0000–0.0062)
Russians (Rostov)		648	9/1296 0.0069 (0.0032–0.0131)	0/1296 0.0000 (0.0000–0.0023)	1/1296 0.0008 (0.0000–0.0043)	0/210 0.0000 (0.0000–0.0149)	0/210 0.0000 (0.0000–0.0149)	1/290 0.0034 (0.0001–0.0019)
Russians (Kirov)		354	4/708 0.0056 (0.0015–0.0144)	0/708 0.0000 (0.0000–0.0042)	0/708 0.0000 (0.0000–0.0042)	0/200 0.0000 (0.0000–0.0149)	0/200 0.0000 (0.0000–0.0149)	0/200 0.0000 (0.0000–0.0149)
Russians (Tver)		182	1/364 0.0027 (0.0001–0.0152)	1/364 0.0027 (0.0001–0.0152)	0/364 0.0000 (0.0000–0.0089)	0/200 0.0000 (0.0000–0.0149)	0/200 0.0000 (0.0000–0.0149)	0/200 0.0000 (0.0000–0.0149)
Russians (Pskov)		140	1/280 0.0036 (0.0001–0.0197)	0/280 0.0000 (0.0000–0.0106)	0/280 0.0000 (0.0000–0.0106)	0/200 0.0000 (0.0000–0.0149)	0/200 0.0000 (0.0000–0.0149)	0/200 0.0000 (0.0000–0.0149)
**Volgo-Ural region**								
Mari	Ural-Yukaghir/Finno-Ugric	505	0/1010 0.0000 (0.0000–0.0030)	0/1010 0.0000 (0.0000–0.0.0030)	0/1010 0.0000 (0.0000–0.0030)	1/380 0.0026 (0.0001–0.0146)	0/300 (0.0000 (0.0000–0.0099)	0/380 0.0000 (0.0000–0.0079)
**(Republic of Mari El)**								
Udmurts (Republic of Udmurtia)	Ural-Yukaghir/Finno-Ugric	613	2/1206 0.0026 (0.0001–0.0146)	0/1206 0.0000 (0.0000–0.0025)	0/1206 0.0000 (0.0000–0.0025)	0/210 0.0000 (0.0000–0.0142)	0/210 0.0000 (0.0000–0.0142)	0/344 0.0000 (0.0000–0.0087)
Chuvash (Republic of Chuvashia)	Altaic/Turkic	780	3/1560 0.0019 (0.0004–0.0056)	1/1560 0.0006 (0.0000–0.0036)	0/1560 0.0000 (0.0000–0.0019)	1/224 0.0045 (0.0001–0.0246)	0/224 0.0000 (0.0000–0.0133)	0/328 0.0000 (0.0000–0.0091)
Bashkirs (Republic of Bashkiria)	Altaic/Turkic	517	0/1034 0.0010 (0.000–0.0031)	1/1034 0.001 (0.0000–0.0054)	0/1034 0.0000 (0.0000–0.0029)	0/510 0.0000 (0.0000–0.0059)	0/510 0.0000 (0.0000–0.0059)	0/534 0.0000 (0.0000–0.0056)
Tatars (Republic of Tatarstan)	Altaic/Turkic	707	14/1414 0.0099 (0.0054–0.0166)	1/1414 0.0007 (0.0000–0.0039)	1/1414 0.0007 (0.0000–0.0039)	3/844 0,0036 (0.0007–0.0104)	4/1414 0.0028 (0.0008–0.0072)	0/400 0.0000 (0.0000–0.0075)
**North Caucasus region**								
Karachay (Republic of Karachay-Cherkessia)	Altaic/Turkic	324	0/648 0.0000 (0.0000–0.0046)	0/648 0.0000 (0.0000–0.0046)	2/648 0.0031 (0.0004–0.0011)	0/648 0.0000 (0.0000–0.0046)	0/648 0.0000 (0.0000–0.0046)	6/648 0.0093 (0.0034–0.0200)
Nogais (Republic of Karachay-Cherkessia)	Altaic/Turkic	118	0/236 0.0000 (0.0000–0.0126)	0/236 0.0000 (0.0000–0.0126)	0/236 0.0000 (0.0000–0.0126)	0/236 0.0000 (0.0000–0.0126)	0/236 0.0000 (0.0000–0.0126)	3/236 0.0127 (0.0026–0.0367)
Circassians (Republic of Karachay-Cherkessia)	North Caucasian/Abkhazian-Adyghe	102	2/204 0.0098 (0.0012–0.0350)	0/204 0.0000 (0.0000–0.0146)	2/204 0.0098 (0.0012–0.0350)	0/204 0.0000 (0.0000–0.0146)	0/204 0.0000 (0.0000–0.0146)	0/204 0.0000 (0.0000–0.0146)
Abaza (Republic of Karachay-Cherkessia)	North Caucasian/Abkhazian-Adyghe	128	1/256 0.0039 (0.0001–0.0216)	0/256 0.0000 (0.0000–0.0116)	3/256 0.0117 (0.0024–0.0339)	0/256 0.0000 (0.0000–0.0116)	0/256 0.0000 (0.0000–0.0116)	1/256 0.0039 (0.0001–0.0216)
Ossetians [Republic of North Ossetia–Alania)]	Indo-European/Iranian	310	1/620 0.0016 (0.0000–0.0090)	0/620 0.0000 (0.0000–0.0048)	0/620 0.0000 (0.0000–0.0048)	0/620 0.0000 (0.0000–0.0048)	0/620 0.0000 (0.0000–0.0048)	2/620 0.0032 (0.0004–0.0116)
Chechens (Republic of Chechnya)	North Caucasian/Nakh-Dagestan	100	0/200 0.0000 (0.0000–0.0149)	0/200 0.0000 (0.0000–0.149)	3/200 0.015 (0.0031–0.0051)	0/200 0.0000 (0.0000–0.0149)	0/200 0.0000 (0.0000–0.0149)	0/200 0.0000 (0.0000–0.0149)

Blood samples were collected during research expeditions in 1995–2018 by the staff of Laboratory of Genetic Epidemiology. The ethnic origin (up to the third generation) was defined by direct interview with examined persons. For this research, all DNA samples studied were anonymized. The DNA was extracted from whole blood samples collected in vacutainers with the preservative EDTA using commercial kits (Wizard Genomic DNA Purification Kit, Promega, United States) according to the manufacturer’s recommendations.

The *CFTR* gene variants in (c.54-5940_273+10250del21kb (p.Ser18ArgfsX16; CFTRdele2,3), c.262_263delTT (p.Leu88 IlefsX22, 394delTT), c.411_412insCTA (p.Leu138dup; L138ins), c.1521_1523delCTT (p.Phe508del, F508del), c.1519_1521 delATC (p.Ile507del, I507del), c.1545_1546delTA (p.Tyr515X; 1677delTA), c.2012delT (p.Leu671X, 2143delT), c.2051_ 2052delAAinsG (p.Lys684SerfsX38, 2183AA>G), c.2052_2053insA (p.Gln685ThrfsX4; 2184insA), c.3691delT (p.Ser1231ProfsX4; 3821delT) by PCR/AFLP (amplified fragment length polymorphism) analysis, variants c.274G>A (p.Glu92Lys, E92K) and c.3846G > A (p.Trp1282X; W1282X) were tested by PCR/RFLP (restriction fragment length polymorphism) analysis according to previously described protocol (Petrova et al., 2008, 2020a; Petrova N. V. et al., 2019). Further, variant designation is given according to the “legacy” nomenclature.

The frequency of identified alleles was calculated according to the formula: *p*_*i*_ = *n*_*i*_/*n*, where *n*_*i*_ is the number of *i*-th alleles, *n* is the sample size (the number of tested chromosomes) (Zhivotovsky, 1991). The Exact method was used to calculate 95% confidence intervals (95% CI) (Clopper and Pearson, 1934). The comparison of the population frequencies of variants in different samples was carried out using the Fisher test or χ^2^-test with Yates correction, according to the generally accepted method (Zhivotovsky, 1991).

Maps of population frequency distribution for variants F508del, 1677delTA, W1282X, and E92K were constructed on the basis of data obtained in our study and on the basis of data on different populations of Europe calculated from the literature sources (Bobadilla et al., 2002; World Health Organization [WHO], 2021) (Supplementary Table 2) using the Bing maps add-in for Excel 365.

## Results

The frequency of eight *CFTR* mutations, CFTRdele2,3, F508del, 1677delTA, 2143delT, 2183AA > G, 2184insA, 394delTT, 3821delT, L138ins, E92K, and W1282X were analyzed in four Russians samples. In Kirov Russians 4 carriers of F508del mutation were found, in Tver Russians – one F508del carrier and one CFTRdele2,3 carrier, in Pskov Russians – one F508del carrier in Rostov Russians – 9 F508del carriers, one 1677delTA carrier and one carrier of W1282X (Table 1).

Population samples of five indigenous peoples of the Volga-Ural region were tested: Mari Udmurts, Bashkirs, Chuvash and Tatars. Only one carrier of E92K variant was found in the sample of Mari people. In the Chuvash and Udmurt samples, three and two carriers of the F508del mutation were found, respectively, and in the Chuvash sample, one carrier of the CFTRdele23 mutation and one carrier of the E92K variant were also found. In the Bashkir sample, the F508del mutation was not detected, but one carrier of the CFTRdele2,3 mutation was detected (Table 1). In the Tatar population, five of the tested variants were identified (Table 1): including the F508del mutation (9 carriers) with the maximum frequency for the indigenous peoples of the Volga-Ural region (0.0099, the frequency differences are significant) (Supplementary Table 3), the L138ins variant was found only in Tatars, and the E92K variant was found in Mari, Chuvash and Tatars.

Six indigenous ethnic populations of the North Caucasus region: two Turkic-speaking (Karachay and Nogais), two Abkhazian-Adyghe peoples (Abaza and Circassians), one Nakh-speaking – Chechens, and Iranian-speaking – Ossetians were studied. In samples from the studied North Caucasus populations carriers of W1282X, F508del and 1677delTA variants were identified. In Karachay, six carriers of W1282X mutation and two of 1677delTA; in Nogais three carriers of W1282X mutation; in Circassians one carrier of F508del and one of 1677delTA mutation; in Abaza – two carriers of F508del, two of W1282X variant, and one carrier of 1677delTA. Chechens had three carriers of 1677delTA variant, Ossetians – one carrier of F508del and two carriers of W1282X (0.0032) (Table 1).

## Discussion

Russians are the most numerous ethnic group in Russia. Up to 80 million ethnic Russians live in the European part of Russian Federation. For the present study four regions with a predominantly Russian population were selected: Pskov, located in the west, Tver – in the center, Rostov – in the south, and Kirov – in the north-east of the European part of Russia. In Russian samples from four regions, four different mutations in the CFTR gene were found: F508del, CFTRdele2, 3 (21kb), 1677delTA, and W1282X, but only F508del was found in all regions, varying in frequency from 0.0027 in the Pskov region to 0.0069 in the Rostov region. The differences in the F508del mutation frequency between Russian samples are not significant (Supplementary Table 4). The average frequency of the F508del mutation in Russians is 0.0056.

**FIGURE 3:**
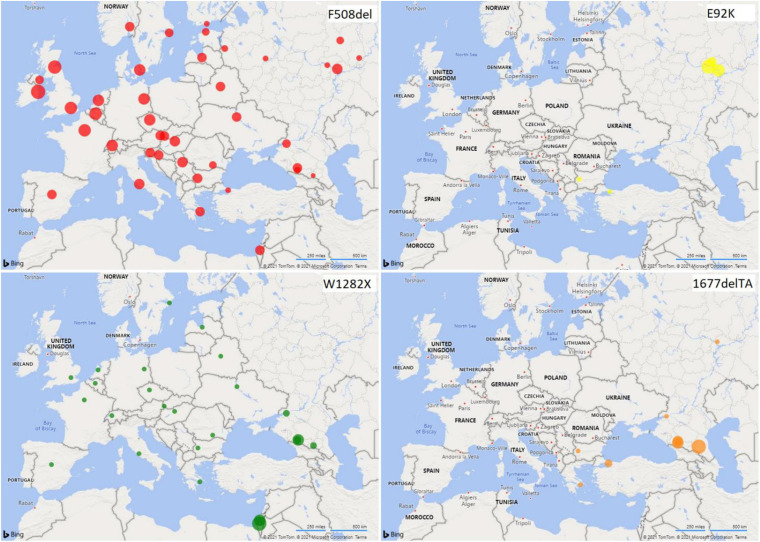
Distribution of *CFTR* variant frequencies in European populations. The radii of the circles reflect the frequency values.

To calculate a more accurate value of the F508del mutation frequency in Russians of the European part of Russia, all samples can be combined. The frequency of F508del revealed in Russians was 0.0056, which is comparable to the data obtained by other researchers studying individuals from Russian populations of central regions of Russia (Abramov et al., 2015), but significantly lower than in a number of European populations (Table 2 and Figure 3). Thus, the highest population frequencies of F508del mutation were observed in the north-west of Western Europe, reaching in Scotland and Denmark – 0.015 and 0.013, respectively (Brandt et al., 1994; Brock et al., 1998); in the Mediterranean countries, the frequency of F508del mutation was lower: for example, in Italy – 0.010 (Gasparini et al., 1999), and in Israel (among Ashkenazi Jews) – 0.0089 (Kalman et al., 1994; Quint et al., 2005; World Health Organization [WHO], 2021). In Estonia, the F508del variant frequency was 0.0059 (Teder et al., 2000), which is not significantly different from the one obtained for Russians in the European part of Russia. The relative frequency of F508del mutation in CF patients decreases from northwestern to southeastern Europe (Bobadilla et al., 2002; Farrell et al., 2018; World Health Organization [WHO], 2021). Apparently, the population frequency of the F508del mutation also changes. This is also consistent with the low frequency of F508del mutation observed in the indigenous population of India (0.00209) (Kapoor et al., 2006).

**TABLE 2:** F508del mutation frequencies in some populations of the world (comparison on Russians of European part of Russia).

**Population (references)**	**No. mutations/no. chromosomes**	**Mutation frequency**	***P*-value**
Russians/European Russia/(Abramov et al., 2015)	15/2,000	0.0075	0.4391
Scotland (Brock et al., 1998)	816/54,322	0.01503	<0.0001
Denmark (Brandt et al., 1994)	172/13,198	0.01303	0.0014
Italy (Gasparini et al., 1999)	90/8,952	0.0101	0.0362
Israel (Jews-Ashkenazi) (Kalman et al., 1994)	35/3,892	0.0089	0.1293
Estonia (Teder et al., 2000)	88/14,792	0.0059	0.8603
India (Kapoor et al., 2006)	4/1,910	0.0021	0.1067

The Volga-Ural region of Russia is situated at the border of Europe and Asia and during historical times was a place of interaction of many ethnic groups (Alekseev, 1974; Kuzeev, 1985).

Three Turkic-speaking groups (Tatars, Chuvash, and Bashkirs) and two Finno-Ugric groups (Mari and Udmurts) were studied. In two of studied Turkic-speaking populations of the Volga-Ural region, F508del mutation was found with relatively high frequencies of 0.0099, 0.0019 in Tatars and Chuvashes, respectively, but was not revealed in Bashkirs. Finno-Ugric populations of the Volga-Ural region demonstrated a low frequency of F508del mutation in Udmurts (0.0016) and its absence in Maries (Table 1). When comparing the frequency of F508del with Russians, the differences were significant only for the Mari and Bashkirs (*p* = 0.0351 and 0.0326) (Supplementary Table 3).

Stepanova et al. (2012) showed that in Chuvash CF patients, the predominant cause of the disease was E92K and F508del variants, the carrier frequency of the E92K mutation is 1: 68 (5/343 persons), and the F508del mutation is 1: 86 (4/343). The differences in the frequencies of these two variants in Stepanova’s work and in our work are not significant (*p* = 1.000 and 0.2630, correspondently). Among the studied Turkic-speaking groups of the Volga-Ural region, variants E92K and F508del were found in the Tatar population, while these variants were not found in the Bashkir population. It should be noted that according to the Russian CF Patients Registry-2018 (RCFPR-2018), the CF incidence in Bashkirs is significantly lower than in Chuvash and Tatars (MEDPRAKTIKA-M, 2019).

The North Caucasus region is characterized by a wide variety of ethnic populations, complicated history of the formation of ethnic groups and high genetic diversity (Alekseev, 1974). The F508del variant was not detected in the Turkic-speaking populations of the North Caucasus (Karachay and Nogais) and in the Chechens: the W1282X variant was predominant in the former, and the 1677delTA variant in the latter (Figure 3). A significant difference between the samples of Ossetians and the samples of Abaza (*p* < 0.05), Chechens (*p* < 0.05), and Circassians (*p* < 0.1) in the 1677delTA variant frequency is shown (Supplementary Table 5). When comparing ethnic groups of the Volga-Ural region and the North Caucasus region, significant differences in the frequency of F508del were found between Karachays and Tatars (*p* < 0.05), as well as between Circassians and Mari (*p* = 0.0243) and Bashkirs (0.0256) (Table 2, Supplementary Table 6, and Figure 3).

The W1282X mutation was assumed to occur as a single mutation event in a population of Middle Eastern Jews before their migration to Europe (World Health Organization). Further distribution of this mutation in various regions was connected with the resettlement of Ashkenazi Jews. The W1282X mutation was found in different regions of the world (Figure 3). The highest frequency of the mutation was found in the population of Ashkenazi Jews (up to 50% of the mutant alleles among CF patients, carrier frequency – 1: 54 and population frequency – 0.0092) (Kalman et al., 1994; Quint et al., 2005). The high population frequency of W1282X mutation was found in Turkic-speaking North Caucasus groups (Karachay and Nogais, 0.0092 and 0.0132), in Abaza (0.0039) and in Ossetians (0.0032). The 1677delTA mutation was previously found to be common in populations neighboring or with historic links to the greater Black Sea region (e.g., Bulgaria, Romania, Greece, Cyprus, and Turkey [Estivill et al., 1997; Bobadilla et al., 2002; Atag et al., 2019; Petrova G. et al., 2019; World Health Organization [WHO], 2021)], including Northern Iran and Georgia (Ivashchenko and Baranov, 2002). We found the high population frequencies of 1677delTA variant in such autochthonous populations of the North Caucasus as Abkhazian-Adyghe [Abaza (0.0171) and Circassians (0.0098)] and Nakh [Chechens (0.0150)] groups, but not in Ossetians and Nogais (Figure 3). Significant differences in the 1677delTA variant frequency were shown in Abkhazian-Adyghe (Circassians, *p* < 0.05; and Abazins, *p* < 0.01) and Nakh groups (Chechens, *p* < 0.01) compared to all studied ethnic groups of Volga-Ural region (Table 2 and Supplementary Table 6). The data obtained in this study allow, to a certain extent, to fill the gap in information on the prevalence of the F508del, E92K, 1677delTA, and W1282X variants of the *CFTR* gene in some indigenous ethnic groups living on the territory of European Russia, and to get an entire picture of the prevalence lapse rate in the considered region. Further studies are necessary to consider the importance of extensive study of the CF pathogenic variants in the populations of the European and North Caucasian part of the Russian Federation, by direct gene sequencing to determine the molecular basis of CF in Russian Federation.

## Data Availability Statement

The raw data supporting the conclusions of this article will be made available by the authors, without undue reservation.

## Ethics Statement

The studies involving human participants were reviewed and approved by the Ethical Committee of Research Centre for Medical Genetics (Research Centre for Medical Genetics, 115522, Moscow, Moskvorechie St., 1, Russian Federation, Protocol No 17/2006 of 02.02.2006). The patients/participants provided their written informed consent to participate in this study.

## Author Contributions

NP: conceptualization and writing – original draft preparation. RZ and VG: resources. NP and NB: investigation. TV: validation. NP and AM: formal analysis. NK: data curation. AM, TV, NK, and EG: writing – review and editing. SK and RZ: project administration and funding acquisition. All authors have read and agreed to the published version of the manuscript.

## Conflict of Interest

The authors declare that the research was conducted in the absence of any commercial or financial relationships that could be construed as a potential conflict of interest.

## Abbreviations

CF: cystic fibrosis
CFTR: cystic fibrosis transmembrane regulator
DNA: deoxyribonucleic acid
PCR: polymerase chain reaction
RFLP: restriction fragment length polymorphism.
