# Supplementary material for: Ethnic Differences in the Frequency of CFTR Gene Mutations in Populations of the European and North Caucasian Part of the Russian Federation
Source: Front Genet. 2021 Jun 16;12:678374. doi: 10.3389/fgene.2021.678374 (PMC8242336; doi:10.3389/fgene.2021.678374)
Supplement: Supplementary file 3 [file Table_3.docx]

**Supplementary Table2. Comparison of the *CFTR* gene variants in populations of Volgo-Ural region (P values are presented).**

|  | Variant frequency | n variant/  n chromosomes | Mari | Udmurts | Chuvash | Bashkirs | Tatars |
| --- | --- | --- | --- | --- | --- | --- | --- |
|  | **F508del** |  |  |  |  |  |  |
| Mari | - | 0/1010 |  |  |  |  |  |
| Udmurts | 0.0016 | 2/1226 | 0.5664 |  |  |  |  |
| Chuvash | 0.0019 | 3/1560 | 0.4219 | 0.8567 |  |  |  |
| Bashkirs | - | 0/1034 |  | 0.5556 | 0.4116 |  |  |
| Tatars | 0.0099 | 14/1414 | 0.0037 | 0.0132 | 0.0083 | 0.0033 |  |
| Russians (total) | 0.0056 | 15/2648 | 0.0351 | 0.1323 | 0.1207 | 0.0326 | 0.1828 |
|  | **E92K** |  |  |  |  |  |  |
| Mari | 0.0026 | 1/380 |  |  |  |  |  |
| Udmurts | - | 0/210 | 1.0000 |  |  |  |  |
| Chuvash | 0.0044 | 1/224 | 1.0000 | 0.3324 |  |  |  |
| Bashkirs | - | 0/510 | 0.4270 |  | 0.6720 |  |  |
| Tatars | 0.0036 | 3/844 | 1.0000 | 0.8875 | 0.8429 | 0.4524 |  |
|  | **L138ins** |  |  |  |  |  |  |
| Mari | - | 0/300 |  |  |  |  | 0.7935 |
| Udmurts | - | 0/210 |  |  |  |  | 0.9757 |
| Chuvash | - | 0/224 |  |  |  |  | 0.9416 |
| Bashkirs | - | 0/510 |  |  |  |  | 0.5218 |
| Tatars | 0.0028 | 4/1414 |  |  |  |  |  |
