# Supplementary material for: Ethnic Differences in the Frequency of CFTR Gene Mutations in Populations of the European and North Caucasian Part of the Russian Federation
Source: Front Genet. 2021 Jun 16;12:678374. doi: 10.3389/fgene.2021.678374 (PMC8242336; doi:10.3389/fgene.2021.678374)
Supplement: Supplementary file 4 [file Table_4.docx]

**Supplementary Table 1.** **Comparison of F508del frequencies in Russian populations (p values are presented).**

| Population (region) | F508del frequency | N mutations/  N chromosomes | Rostov | Kirov | Tver |
| --- | --- | --- | --- | --- | --- |
| Rostov | 0.0069 | 9/1296 |  |  |  |
| Kirov | 0.0056 | 4/708 | 0.9569 |  |  |
| Tver | 0.0027 | 1/364 | 0.5954 | 0.8515 |  |
| Pskov | 0.0036 | 1/280 | 0.8184 | 0.6782 | 0.8522 |
