# Supplementary material for: Ethnic Differences in the Frequency of CFTR Gene Mutations in Populations of the European and North Caucasian Part of the Russian Federation
Source: Front Genet. 2021 Jun 16;12:678374. doi: 10.3389/fgene.2021.678374 (PMC8242336; doi:10.3389/fgene.2021.678374)
Supplement: Supplementary file 5 [file Table_5.docx]

**Supplementary Table3**. **Comparison of the *CFTR* gene variants in populations of North Caucasus region (p values are presented).**

| Population | Variant frequence | n variant /  n chromo  somes | Karachay | Nogai | Circassians | | Abaza | Ossetians | Chechens |
| --- | --- | --- | --- | --- | --- | --- | --- | --- | --- |
|  | **F508del** |  |  |  |  |  | |  |  |
| Karachay |  | 0/648 |  |  |  |  | |  |  |
| Nogai |  | 0/236 | - |  |  |  | |  |  |
| Circassians | 0.0098 | 2/204 | 0.0571 | 0.2144 |  |  | |  |  |
| Abaza | 0.0039 | 1/256 | 0.2832 | 0.9238 | 0.8433 |  | |  |  |
| Ossetians | 0.0016 | 1/620 | 0.4890 | 0.3811 | 0.3102 | 0.5178 | |  |  |
| Chechens |  | 0/200 | - | - | 0.4871 | 0.3762 | | 0.5698 |  |
| Russians(all) | 0.0056 | 15/2648 | 0.1108 | 0.4921 | 0.7886 | 0.7166 | | 0.3264 | 0.5750 |
|  | **1677delTA** |  |  |  |  |  | |  |  |
| Karachay | 0.0031 | 2/648 |  |  |  |  | |  |  |
| Nogai |  | 0/236 | 0.9567 |  |  |  | |  |  |
| Circassians | 0.0098 | 2/204 | 0.5242 | 0.2144 |  |  | |  |  |
| Abaza | 0.0117 | 3/256 | 0.2806 | 0.2497 | 0.8508 |  | |  |  |
| Ossetians |  | 0/620 | 0.4987 | - | 0.0611 | 0.0248 | |  |  |
| Chechens | 0.0150 | 3/200 | 0.1629 | 0.0957 | 0.6829 | 1.0000 | | 0.0143 |  |
|  | **W1282X** |  |  |  |  |  | |  |  |
| Karachay | 0.0092 | 6/648 |  |  |  |  | |  |  |
| Nogai | 0.0127 | 3/236 | 0.7070 |  |  |  | |  |  |
| Circassians |  | 0/204 | 0.3448 | 0.2523 |  |  | |  |  |
| Abaza | 0.0039 | 1/256 | 0.6800 | 0.3543 | 1.0000 |  | |  |  |
| Ossetians | 0.0032 | 2/620 | 0.2888 | 0.1318 | 1.0000 | 1.0000 | |  |  |
| Chechens |  | 0/200 | 0.3450 | 0.2534 |  | 1.0000 | | 1.000 |  |
| Jews-Ashkenazi [[Kalman](https://pubmed.ncbi.nlm.nih.gov/?term=Kalman+YM&cauthor_id=8044659) Y M, 1994] | 0.0092 | 36/3892 | 0.9981 | 0.8514 | 0.3197 | 0.5908 | | 0.1978 | 0.3281 |
