# Supplementary material for: Ethnic Differences in the Frequency of CFTR Gene Mutations in Populations of the European and North Caucasian Part of the Russian Federation
Source: Front Genet. 2021 Jun 16;12:678374. doi: 10.3389/fgene.2021.678374 (PMC8242336; doi:10.3389/fgene.2021.678374)
Supplement: Supplementary file 6 [file Table_6.docx]

## Supplementary Table 4. Comparison of the *CFTR* gene variants in ethnic populations of Volga-Ural and North Caucasus regions and ethnic Russians from European part of Russian Federation (p values are presented).

| Population | Variant frequency | n variant /  n chromo  somes | Mari | Udmurts | Chuvash | | Bashkirs | Tatars |  |
| --- | --- | --- | --- | --- | --- | --- | --- | --- | --- |
|  | **F508del** |  |  |  |  |  | |  |  |
|  |  |  | - | 0.0016 | 0.0019 | - | | 0.0099 |  |
|  |  |  | 0/1010 | 2/1226 | 3/1560 | 0/1034 | | 14/1414 |  |
| Karachay |  | 0/648 | - | 0.7757 | 0.6293 | - | | 0.0243 |  |
| Nogai |  | 0/236 | - | 0.5347 | 0.5002 | - | | 0.2494 |  |
| Circassians | 0.0098 | 2/204 | 0.0276 | 0.1833 | 0.1968 | 0.0256 | | 0.9895 |  |
| Abaza | 0.0039 | 1/256 | 0.2022 | 0.4341 | 0.4557 | 0.1984 | | 0.4918 |  |
| Ossetians | 0.0016 | 1/620 | 0.3804 | 0.9926 | 0.8787 | 0.7959 | | 0.0837 |  |
| Chechens |  | 0/200 | - | 0.5676 | 0.5348 | - | | 0.3144 |  |
| Russians(all) | 0.0056 | 15/2648 | 0.0351 | 0.1323 | 0.1207 | 0.0326 | | 0.1828 |  |
|  | **1677delTA** |  |  |  |  |  | |  |  |
|  |  |  | - | - | - | - | | 0.0007 |  |
|  |  |  | 0/1010 | 0/1226 | 0/1560 | 0/1034 | | 1/1414 |  |
| Karachay | 0.0031 | 2/648 | 0.2976 | 0.2292 | 0.1561 | 0.2889 | | 0.4880 |  |
| Nogai |  | 0/236 | - | - | - | - | | 0.6828 |  |
| Circassians | 0.0098 | 2/204 | 0.0276 | 0.0140 | 0.0050 | 0.0256 | | 0.0435 |  |
| Abaza | 0.0117 | 3/256 | 0.0082 | 0.0051 | 0.0028 | 0.0077 | | 0.0126 |  |
| Ossetians |  | 0/620 | - | - | - | - | | 0.5078 |  |
| Chechens | 0.0150 | 3/200 | 0.0045 | 0.0027 | 0.0014 | 0.0016 | | 0.0023 |  |
| Russians(all) | 0.0004 | 1/2648 | 0.5368 | 0.4963 | 0.4428 | 0.5321 | | 0.6518 |  |
|  | **W1282X** |  |  |  |  |  | |  |  |
|  |  |  | 0/380 | 0/344 | 0/328 | 0/534 | | 0/400 |  |
| Karachay | 0.0092 | 6/648 | 0.1451 | 0.1738 | 0.1886 | 0.0691 | | 0.1314 |  |
| Nogai | 0.0127 | 3/236 | 0.1078 | 0.1317 | 0.1441 | 0.0474 | | 0.0967 |  |
| Circassians |  | 0/204 | - | - | - | - | | - |  |
| Abaza | 0.0039 | 1/256 | 0.8423 | 0.8820 | 0.9010 | 0.7068 | | 0.8218 |  |
| Ossetians | 0.0032 | 2/620 | 0.7046 | 0.7522 | 0.7751 | 0.5459 | | 0.6802 |  |
| Chechens |  | 0/200 | - | - | - | - | | - |  |
| Russians(all) | 0.0011 | 1/890 | 0.5133 | 0.5340 | 0.5436 | 0.4384 | | 0.5024 |  |
